# Revisiting the Role of Insulin-Like Growth Factor-I Receptor Stimulating Activity and the Apolipoprotein E in Alzheimer’s Disease

**DOI:** 10.3389/fnagi.2019.00020

**Published:** 2019-02-12

**Authors:** Sara A. Galle, Ashley van der Spek, Madeleine L. Drent, Michael P. Brugts, Erik J. A. Scherder, Joseph A. M. J. L. Janssen, M. Arfan Ikram, Cornelia M. van Duijn

**Affiliations:** ^1^Department of Clinical, Neuro- and Developmental Psychology, Vrije Universiteit Amsterdam, Amsterdam, Netherlands; ^2^Department of Genetic Epidemiology, Erasmus Medical Center, Rotterdam, Netherlands; ^3^Section of Endocrinology, Department of Internal Medicine, Amsterdam University Medical Center, Amsterdam, Netherlands; ^4^Department of Internal Medicine, Ikazia Ziekenhuis, Rotterdam, Netherlands; ^5^Department of Internal Medicine, Erasmus Medical Center, Rotterdam, Netherlands; ^6^Department of Epidemiology, Erasmus Medical Center, Rotterdam, Netherlands; ^7^Department of Neurology, Erasmus Medical Center, Rotterdam, Netherlands; ^8^Department of Radiology, Erasmus Medical Center, Rotterdam, Netherlands; ^9^Nuffield Department of Population Health, Big Data Institute, Li Ka Shing Centre for Health Information and Discovery, University of Oxford, Oxford, United Kingdom

**Keywords:** Alzheimer’s disease, dementia, genetic epidemiology, insulin-like growth factor I, KIRA assay, apolipoprotein E

## Abstract

**Background**: Alterations in insulin-like growth factor I (IGF-I) signaling have been associated with dementia and Alzheimer’s disease (AD). Studies on the association between IGF-I levels and dementia risk have been inconclusive. We reported earlier that higher levels of IGF-I receptor stimulating activity are associated with a higher prevalence and incidence of dementia.

**Objective**: In the present study, we test the robustness of the association between IGF-I receptor stimulating activity and dementia by extending the follow-up period to 16 years and investigate possible effect modification by apolipoprotein E (ApoE).

**Methods**: At baseline, circulating IGF-I receptor stimulating activity was determined by the IGF-I kinase receptor activation (KIRA) assay in 1,014 elderly from the Rotterdam Study. Dementia was assessed from baseline (1997–1999) to follow-up in January 2015. Associations of IGF-I receptor stimulating activity and incident dementia were assessed with Cox proportional hazards models.

**Results**: During 10,752 person-years of follow-up, 174 people developed dementia. In the extended follow-up we no longer observed a dose-response relationship between IGF-I receptor stimulating activity and risk of dementia [adjusted odds ratio 1.11; 95% confidence interval (CI) 0.97–1.28]. Interestingly, we found evidence of an interaction between ApoE-ε4 and tertiles of IGF-I receptor stimulating activity. IGF-I receptor stimulating activity in the median and top tertiles was related to increased dementia incidence in hetero- and homozygotes of the ApoE-ε4 allele, but did not show any association with dementia risk in people without the ApoE-ε4 allele (adjusted odds ratio medium vs. low IGF-I receptor stimulating activity in ApoE-ε4 carriers: 1.45; 95% CI 1.00–2.12). These findings suggest a threshold effect in ApoE-ε4 carriers. In line with the hypothesis that downregulation of IGF-I signaling is associated with increased dementia risk, ApoE-ε4 homozygotes without prevalent dementia displayed lower levels of IGF-I receptor stimulating activity than heterozygotes and non-carriers.

**Conclusion**: The findings shed new light on the association between IGF-I signaling and the neuropathology of dementia and ask for replication in other cohorts, using measures of IGF-I receptor stimulating activity rather than total serum levels as putative markers of dementia risk.

## Introduction

Insulin-like growth factor I (IGF-I) is a multifunctional peptide hormone known to modulate multiple cellular processes including proliferation, differentiation, energy metabolism, glucose homeostasis, stress resistances and apoptosis. Downregulation of IGF-I signaling is found in the elderly and in patients with type 2 diabetes. In contrast, elevated concentrations of circulating IGF-I have been associated with an increased risk of prostate, breast (Hankinson et al., 1998; Renehan et al., 2004; Kaaks, 2008), colorectal (Ma et al., 1999; Wu et al., 2002; Kaaks, 2008) and lung (Yu et al., 1999) cancer. IGF-I signaling is also markedly disturbed in the brain of patients with Alzheimer’s Disease (AD; Frölich et al., 1998, 1999; Moloney et al., 2010) with alterations in both the levels and phosphorylation state of IGF-I receptor (IGF-IR) as well as the levels and distribution of IGF-I and IGF-IR mRNA in the brain (Rivera et al., 2005; Steen et al., 2005). Dysregulation progresses as the disease advances (Ostrowski et al., 2016). It remains unclear whether alterations in IGF-I signaling are a causal factor in the pathogenesis of AD or rather a consequence. Findings of experimental and observational studies have been controversial.

In experimental studies, reduced IGF-I signaling has been linked to increased amyloid β (Aβ) deposition (Carro et al., 2002; Ashpole et al., 2015), development of phosphorylated tau (Gasparini et al., 2001, 2002; Cheng et al., 2005), increased oxidative stress, neuro-inflammation and apoptosis (Bedse et al., 2015). IGF-I can increase the transport of Aβ carrier proteins albumin and transthyretin into the brain. Upon systemic administration, brain levels of albumin and transthyretin increased and the fraction of Aβ bound to carrier proteins in the CSF and blood was elevated. Suggesting that IGF-I reduced brain Aβ load, in part by enhancing its clearance through carrier proteins such as albumin and transthyretin (Carro et al., 2002). Systemic administration of IGF-I has also been shown to lower the toxicity of Aβ in wild type mice (Aguado-Llera et al., 2005) and restore cognitive function in mouse models of AD (Carro et al., 2006), supporting the potential of IGF-I as a therapeutic target in human patients. Peripheral administration has, however, failed to alter Aβ levels in trials with transgenic rats, mice and dogs (Lanz et al., 2008; Sevigny et al., 2008; Parrella et al., 2013; Trueba-Sáiz et al., 2013). In contrast to the neuroprotective role of IGF-I, it has also been suggested that the downregulation of IGF-I signaling attenuates the effects of aging and neurodegeneration. Suppression of IGF-I signaling has been associated with longevity in humans (Suh et al., 2008) and has shown to delay the process of aging and increase lifespan in model organisms (Tatar et al., 2001; Tazearslan et al., 2011; Milman et al., 2014). In AD mouse models long term suppression of IGF-IR signaling has been linked to reduced neuronal loss, greater resistance to oxidative stress, neuro-inflammation and Aβ aggregation, and has been associated with prolonged preservation of spatial memory and a reduction of behavioral deficits, even when plasma Aβ levels increased (Cohen et al., 2009; Freude et al., 2009; Gontier et al., 2015; George et al., 2017). Last but not least, lowering serum IGF-I *via* a protein restriction diet ameliorated Alzheimer pathology in transgenic mouse models (Parrella et al., 2013).

In human observational studies the role of IGF-I signaling in the risk of AD and dementia remains open to question. Longitudinal analyses of the cumulative dementia incidence in 3,582 participants of the Framingham Heart Study, spanning middle and old age, indicated that for those with the lowest levels of serum IGF-I at baseline dementia risk was increased by 51% (Westwood et al., 2014). No such relation was found by Green et al. (2014) examining the prospective association between total IGF-I, IGF-II, and IGF-I Binding Protein 3 (IGFBP-3) and cognitive function in 724 males participating in the Caerphilly Prospective Study. In this study, both total serum IGF-II and IGFBP-3 were associated with age-related cognitive decline and cognitive impairment, but previous associations of total serum IGF-I with cognitive decline and dementia were not replicated. Correspondingly, a meta-analysis of epidemiological studies on the association between total serum IGF-I and dementia nullified the results of previous studies. Five studies suggested that increased levels of circulating total IGF-I predict a higher risk of AD, while three studies suggested an inverse association and two studies reported no significant differences between groups (Ostrowski et al., 2016). Differences in findings across studies are speculatively attributed to differences in age of onset and stage of disease progression, comorbid diabetes, or the differential influence of IGF-I gene polymorphisms. Although, the majority of studies report a contribution of alterations in IGF-I signaling to the prediction of dementia risk independent of apolipoprotein E *(ApoE)* genotype (Vargas et al., 2011; Talbot et al., 2012; van Exel et al., 2014; Lane et al., 2017), Deelen et al. (2011) reported an association between the ApoE-ε4 allele and lowered total serum IGF-I levels in middle-aged women. However, a recent Mendelian randomization study by Williams et al. (2018) did not provide any evidence for an association between genetically predicted variation in total IGF-I or its binding protein IGFBP-3 and risk of AD. These findings decrease the probability that total serum IGF-I is the relevant determinant of AD and dementia.

As most of the circulating IGF-I measured in serum is bound to IGF-I binding proteins and therefore biologically inactive, levels of total IGF-I poorly reflect the actual IGF-I bioactivity. We therefore applied an IGF-I specific kinase receptor activation assay (KIRA) to assess IGF-I bioactivity, by measuring IGF-I receptor stimulating activity (Chen et al., 2003; Brugts et al., 2010). IGF-I receptor stimulating activity takes into account the modifying effect of IGF-I binding proteins on the interaction between IGF-I and the IGF-I receptor and measures the net effects on IGF-I receptor activation. In a previous study we have shown that IGF-I bioactivity is positively related to total and free IGF-I levels obtained by IGF-I immunoassays. Interestingly, correlations were relatively weak (0.52 for total IGF-I and 0.20 for free IGF-I respectively), suggesting that the IGF-I KIRA assay produces new information about IGF-I signaling (Brugts et al., 2008).

We reported earlier that higher levels of IGF-I receptor stimulating activity were associated with a higher prevalence and a higher incidence of dementia (de Bruijn et al., 2014). In light of the conflicting results of the experimental and human studies, we aimed to test the long-term robustness of the association between IGF-I receptor stimulating activity and dementia risk by extending the follow up period with another 4 years and investigate possible effect modification by *ApoE*, the major genetic driver of AD and dementia risk.

## Materials and Methods

### Setting

This study was embedded within the prospective, population-based Rotterdam Study, designed to study risk factors and determinants of chronic diseases in the elderly population. The Rotterdam Study began in 1990, with an invitation to inhabitants of 55 years and older residing in Ommoord, a district of Rotterdam in the Netherlands. Of the 10,215 people invited, 7,983 agreed to participate in the examinations at baseline. Up until 2015, there have been five follow-up examinations. Details of the study are described elsewhere (Ikram et al., 2017). Because IGF-I receptor stimulating activity was measured in blood samples collected at the second follow-up examination, between 1997 and 1999, this visit was used as baseline for the current study. Of the 5,990 participants that were alive in 1997–1999, 4,797 persons participated in the second follow-up assessment. IGF-I receptor stimulating activity levels were measured in blood samples of 1,050 randomly selected participants due to financial constraints. Five participants were excluded because their blood samples could not be correctly matched and 14 participants were excluded because measurements did not pass prior defined assay acceptance criteria (inter-assay coefficient of variation <10%). Another 17 participants were excluded because dementia screening was incomplete. Eventually, 1,014 participants were included in the analyses. The Rotterdam Study has been approved by the Medical Ethics Committee of the Erasmus MC (registration number MEC 02.1015) and by the Dutch Ministry of Health, Welfare and Sport (Population Screening Act WBO, license number 1071272-159521-PG). The Rotterdam Study has been entered into the Netherlands National Trial Register (NTR^1^) and into the WHO International Clinical Trials Registry Platform (ICTRP^2^) under shared catalog number NTR6831. All participants provided written informed consent to participate in the study and to have their information obtained from treating physicians.

### Assessment of IGF-I Receptor Stimulating Activity

IGF-I receptor stimulating activity levels were measured using an IGF-I KIRA (intra- and inter-assay coefficients of variation of 5.2 and 12.2%, respectively; cross-reactivity of 15% for IGF-II; Chen et al., 2003; Brugts et al., 2008). Details of the assessment are described previously (de Bruijn et al., 2014).

### Assessment of Dementia

Participants were screened for dementia at baseline and follow-up examinations using a 3-step protocol (Ott et al., 1998). First, screening was performed using the Mini-Mental State Examination (MMSE; Folstein et al., 1975) and the Geriatric Mental Schedule (GMS) organic level (Copeland et al., 1976). People with a MMSE score lower than 26 or GMS organic level higher than 0 were subsequently subjected to further examination and informant interview including the Cambridge Examination for Mental Disorders in the Elderly (CAMDEX; Roth et al., 1986). When necessary, participants underwent further neuropsychological assessment. When information on neuro-imaging was available, it was used as an aid for decision-making. For all suspected cases of dementia, the diagnosis was made by a consensus panel, led by a neurologist. During follow-up the cohort was under continuous surveillance for dementia incidence through electronic linkage of the database of the Rotterdam Study with medical records from general practitioners and the regional institute for outpatient mental health care (de Bruijn et al., 2015). The applied criteria for the diagnosis of dementia and probable AD are in accordance with the standard criteria for dementia (Diagnostic and Statistical Manual of Mental Disorders III-revised; American Psychiatric Association, 1987) and AD (National Institute of Neurological and Communicative Disorders and Stroke and the AD and Related Disorders Association; McKhann et al., 2011). The total cohort was continuously monitored for incidence of dementia through linkage to the digitized medical records from general practitioners and the Regional Institute for Outpatient Mental Health Care. Follow-up for incident dementia is complete until January 2015.

### Other Measurements

Information on *ApoE* genotype was obtained using polymerase chain reaction on coded DNA samples. ApoE-ε4 carrier status was defined as carrier of one or two ε4 alleles. Blood pressure was calculated as the average of two measurements at the right brachial artery using a random-zero sphygmomanometer. Hypertension was defined as a blood pressure ≥140/90 mmHg or use of blood pressure lowering medication, prescribed for the indication of hypertension. Waist circumference was measured in centimeters. Serum glucose, total cholesterol, and high-density lipoprotein (HDL)-cholesterol levels were acquired by an automated enzymatic procedure (Boehringer Mannheim System). Missing values in covariates (for ApoE-ε4 carrier status 4.8%, for all other covariates less than 3.5%) were imputed based on age and sex.

### Statistical Analyses

We examined the association between IGF-I receptor stimulating activity and incident dementia using Cox proportional hazards models. IGF-I receptor stimulating activity was entered per standard deviation (SD) into the models. We also studied IGF-I receptor stimulating activity in tertiles, using the lowest tertile as reference. All models were adjusted for age and sex (Model I) and additionally for hypertension, glucose, waist circumference, ApoE-ε4 carrier status, total cholesterol, and HDL-cholesterol (Model II) for being potential confounders. To investigate possible effect modification by ApoE, the (multiplicative) interaction between ApoE-ε4 carrier status and IGF-I receptor stimulating activity on dementia risk was tested using interaction terms and separate analyses on data stratified on ApoE-ε4 carrier status were performed. The underlying time-scale in the Cox proportional hazards models was the follow-up time, which was defined from time at blood sample collection (1997–1999) until the end of December 2015. Participants were censored within this time period when they were diagnosed with dementia, died, or decided to terminate their participation in the study. We separately investigated the association between IGF-I receptor stimulating activity and AD. Analyses were performed using IBM SPSS statistics version 24.0 (IBM Corp, Armonk, NY, USA).

## Results

Baseline characteristics of the study population are provided in Table 1. At baseline, 31 participants suffered from prevalent dementia, of which 23 had AD. During a follow-up of 10,752 person-years (mean follow-up of 11 years, SD 5.2 years), 174 participants developed dementia, of whom 140 were diagnosed with AD.

**Table 1:** Baseline characteristics.

	Prevalent dementia *N* = 30	At risk for incident dementia *N* = 984
Age, years	81.54 (8.33)	72.04 (7.1)
Females	70%	55.8%
IGF-IRSA, pmol/L	208.13 (77.59)	179.06 (55.48)
Apolipoprotein E-ε4 carrier status	71.4%	27.4%
Hypertension	82.1%	75.5%
Waist circumference, cm	94.17 (8.33)	93.84 (11.1)
Glucose, mmol/L	6.03 (1.07)	6.01 (1.51)
Total cholesterol, mmol/L	5.52 (1.17)	5.83 (1)
HDL-cholesterol, mmol/L	1.34 (0.46)	1.38 (0.37)

*Data are presented as means (standard deviations) or percentages. N, number of people; IGF-IRSA, insulin-like growth factor I receptor stimulating activity; HDL, high-density lipoprotein*.

In the overall proportional hazard analyses, there was no statistically significant evidence for a relation between the level of IGF-I receptor stimulating activity at baseline and risk of dementia. However, the hazard ratio (HR) per SD increase in IGF-I receptor stimulating activity [1.11; 95% confidence interval (CI) 0.97–1.28; see Table 2] was very similar to the HR reported in our previous analyses (1.15; 95% CI 1.00–1.33) with shorter follow-up (de Bruijn et al., 2014). A congruent HR was found for the incidence of AD [HR 1.10 (95% CI 0.95–1.28)].

**Table 2:** IGF-I receptor stimulating activity and risk of incident dementia.

	Dementia	Alzheimer’s disease
	HR (95% CI)	HR (95% CI)
	n/N 174/973	n/N 140/973
Model I	1.09 (0.95–1.25)	1.07 (0.92–1.25)
Model II	1.11 (0.97–1.28)	1.10 (0.95–1.28)

*Data are presented as hazard ratios (HR) and 95% confidence intervals (CI). N, number of people at risk for incident dementia; n, number of cases of incident dementia. IGF IRSA, insulin-like growth factor I receptor stimulating activity. Model I: adjusted for age and sex. Model II: adjusted for age, sex, hypertension, glucose, waist circumference, Apolipoprotein E-ɛ4 status, total cholesterol, and HDL cholesterol*.

**Figure 1:**
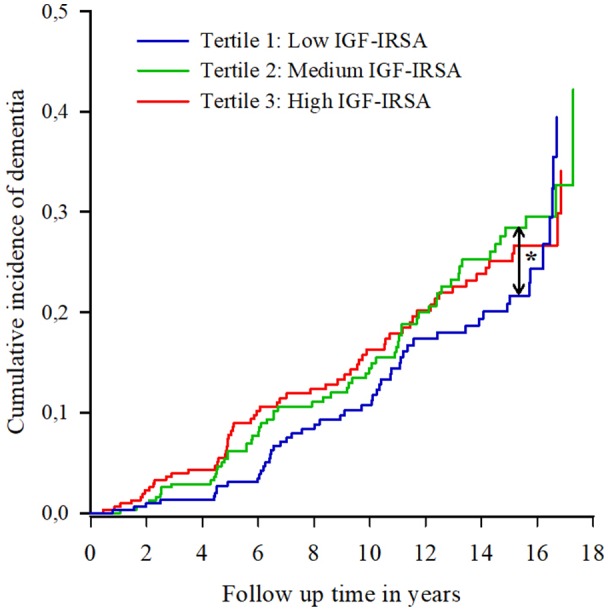
Cumulative incidence curves of dementia per tertile of insulin-like growth factor (IGF-I) receptor stimulating activity (IGF-IRSA). Tertile 1 represents the lowest levels of IGF-I receptor stimulating activity, tertile 2 medium levels and tertile 3 the highest levels. *indicates significant at *p* < 0.05.

Figure 1 shows the cumulative incidence curves of dementia per tertile of IGF-I receptor stimulating activity. Proportional hazard analyses of dementia incidence revealed that those in the lowest tertile of IGF-I receptor stimulating activity at baseline had the lowest risk of dementia [HR moderate vs. low: 1.45 (95% CI 1.00–2.12); HR high vs. low 1.40 (95% CI 0.96–2.04)], while there was no difference in risk of dementia between the medium and highest tertiles (Figure 1).

### IGF-I Receptor Activity and ApoE-ε4

At baseline there was a statistically significant difference in IGF-I receptor stimulating activity between ApoE-ε4 genotype groups without dementia (non-carrier, heterozygote and homozygote), after adjustment for age and sex (*p* = 0.04; see Table 3). The levels of IGF-I receptor stimulating activity were significantly lower in homozygotes for ApoE-ε4 than in people with no copies of the ApoE-ε4 allele (*p* = 0.04). There were no statistically significant differences in level of IGF-I receptor stimulating activity between non-carriers and ApoE-ε4 heterozygotes or ApoE-ε4 heterozygotes and homozygotes.

**Table 3:** IGF-I receptor stimulating activity stratified by apolipoprotein E (ApoE) group.

	Non-carriers *N* = 680	ApoE-ε4 heterozygotes *N* = 240	ApoE-ε4 homozygotes *N* = 17	P for trend *N* = 937
IGF-IRSA (pmol/L)	181.92 (58.58)	174.31 (48.24)	151.82 (34.98)	F(2) 3.20, *p* = 0.04

*Data are presented as means (standard deviations). N, number of persons; IGF-IRSA, insulin-like growth factor I receptor stimulating activity. Adjusted for age and sex*.

When testing for effect modification, significant evidence for a multiplicative interaction between IGF-I receptor stimulating activity and ApoE-ε4 carrier status was observed (*χ*^2^(2) = 10.85, *p* = 0.004). In those without the ApoE-ε4 variant, the level of IGF-I receptor stimulating activity was not associated with the risk of dementia (medium vs. low: HR 0.97 (95% CI 0.59–1.60); high vs. low: HR 1.09 (95% CI 0.67–1.77); *χ*^2^(2) = 0.24, *p* = 0.89). For those with one or more copies of the ApoE-ε4 allele, level of IGF-I receptor stimulating activity was positively associated with dementia risk. Dementia risk was significantly increased in people with one or more copies of the ApoE-ε4 allele and IGF-1 receptor stimulating activity in the median and top tertiles compared to those with IGF-I receptor stimulating activity in the bottom tertile [medium vs. low: HR 3.80 (95% CI 1.90–7.60); high vs. low: HR 2.71 (95% CI 1.37–5.38)].

**Figure 2:**
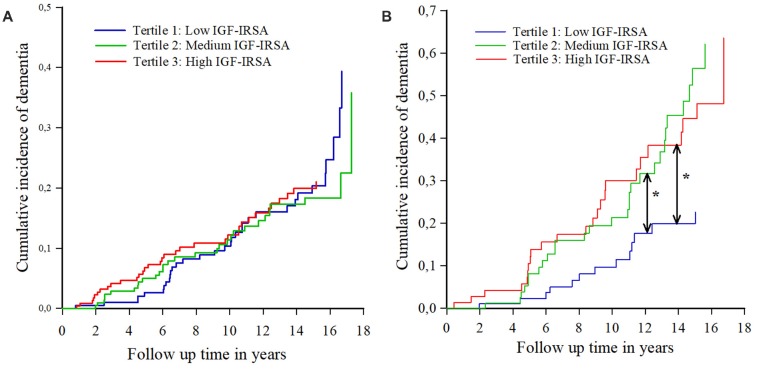
**(A)** Cumulative incidence curves of dementia per tertile of IGF-I receptor stimulating activity (IGF-IRSA), for persons without apolipoprotein E ε4 allele (ApoE-ε4). Tertile 1 represents the lowest levels of IGF-I receptor stimulating activity, tertile 2 medium levels and tertile 3 the highest levels. **(B)** Cumulative incidence curves of dementia per tertile of IGF-I receptor stimulating activity (IGF-IRSA), for hetero- and homozygotes of the ApoE-ε4 allele. Tertile 1 represents the lowest levels of IGF-I receptor stimulating activity, tertile 2 medium levels and tertile 3 the highest levels. *Indicates significant at *p* < 0.05.

Similar results were found for the incidence of AD (Table 4). Figures 2A,B show the cumulative incidence of dementia per tertile group of IGF-I receptor stimulating activity, stratified by ApoE-ε4 genotype.

**Table 4:** IGF-I receptor stimulating activity tertile groups and risk of incident dementia.

	Dementia		Alzheimer’s disease	
	ApoE–ε4+	ApoE–ε4−	ApoE–ε4+	ApoE–ε4−
	n/N 65/255	n/N 97/669	n/N 51/255	n/N 78/669
IGF-IRSA groups	HR (95% CI)	HR (95% CI)	HR (95% CI)	HR (95% CI)
Medium	3.80 (1.90–7.60)	0.97 (0.59–1.60)	3.44 (1.61–7.34)	1.00 (0.57–1.74)
High	2.71 (1.37–5.38)	1.09 (0.67–1.77)	2.38 (1.12–5.08)	1.01 (0.86–1.75)
Medium vs. High	1.40 (0.79–2.48)	0.89 (0.54–1.46)	1.44 (0.75–2.76)	0.99 (0.57–1.73)

*Data are presented as hazard ratios (HR) and 95% confidence intervals (CI). N, number of people at risk for incident dementia. n, number of cases of incident dementia. IGF-IRSA, insulin-like growth factor I receptor stimulating activity divided in tertile groups. The lowest tertile group is used as reference. Adjusted for age, sex, hypertension, glucose, waist circumference, total cholesterol, and HDL cholesterol*.

## Discussion

In the extended follow-up period of 16 years, our study did not find evidence for a long-term dose response association between circulating IGF-I receptor stimulating activity at baseline and the future risk of dementia. Interestingly, we found evidence of an interaction between ApoE-ε4 and IGF-I receptor stimulating activity. In those hetero- and homozygous for the ApoE-ε4 allele, dementia risk was increased in persons with medium and high levels of IGF-I receptor stimulating activity at baseline, compared to those with low IGF-I receptor stimulating activity at baseline. However, no relation between IGF-I receptor stimulating activity and dementia risk was observed in non-carriers of the ApoE-ε4 allele. This suggests that, in ApoE-ε4 carriers, there is a certain threshold above which IGF-I receptor stimulating activity becomes associated with dementia at long-term follow up. In addition, in individuals without dementia, IGF-I receptor stimulating activity was lower in homozygote carriers of ApoE-ε4 than in people with other ApoE genotypes.

To our knowledge the Rotterdam Study is still the only study that has investigated the role of circulating IGF-I receptor stimulating activity in relation to dementia. As circulating IGF-I receptor stimulating activity is only modestly correlated to total serum IGF-I and IGF-I/IGFBP-3 ratio (Brugts et al., 2008), comparison to other studies on serum total IGF-I, measured by immunoassays, and dementia risk, described by Ostrowski et al. (2016) is difficult.

We found a modifying effect of ApoE-ε4 on circulating IGF-I receptor stimulating activity at baseline and an interaction on the relation between IGF-I receptor stimulating activity and the risk of dementia. The observed interaction between circulating IGF-I receptor stimulating activity and ApoE isoforms in our study could reflect opposing influences on shared pathways involved in Alzheimer pathology. Both ApoE and IGF-I are involved in the regulation of AD biomarkers: IGF-I is an important mediator in the clearance and regulation of Aβ in the brain, enhances survival of neurons exposed to Aβ and inhibits tau phosphorylation (Doré et al., 1997; Carro et al., 2002, 2006; Cheng et al., 2005; Engel et al., 2006; Moloney et al., 2010; Talbot et al., 2012). The ApoE-ε4 allele, on the other hand, is associated with decreased Aβ_1–42_ and higher tau and p-tau in the CSF and increased cerebral amyloid deposition across the AD spectrum (Tapiola et al., 2000; Morris et al., 2010; Leoni, 2011; Risacher and Saykin, 2013; Wildsmith et al., 2013; Kanekiyo et al., 2014). In mice expressing human ApoE-ε4, increased tau phosphorylation has been demonstrated (Tesseur et al., 2000). ApoE-ε4 and IGF-I also have an opposing influence on NMDA receptor signaling. The NR2B subunit of the NMDA receptor, in particular, is suggested to be of specific importance for spatial learning and long-term potentiation, impaired in AD (Sonntag et al., 2000; Le Grevès et al., 2005; Reiman et al., 2009). The ApoE-ε4 genotype is associated with decreased NR2B NMDA receptor subunit levels and enhances age-related decline in cognitive function by down-regulating signaling in mice (Liu et al., 2015). In contrast, IGF-I positively affects the NMDA receptor pathway by increasing the NR2B subunit mRNA transcript of the hippocampal NMDA receptor in rats (Sonntag et al., 2000; Le Grevès et al., 2005). The observed association between elevated levels of IGF-I receptor stimulating activity and increased risk of dementia in ApoE-ε4 carriers might thus be a reflection of a compensatory response to neuropathological changes associated with the ApoE-ε4 genotype and a preclinical loss of sensitivity of the IGF-I receptor.

The strengths of our study are its prospective, population-based design, the long follow-up period, and the use of a direct measure of circulating IGF-I receptor stimulating activity. However, there are also some limitations. First, IGF-I receptor stimulating activity was only measured in peripheral blood samples. Even though circulating IGF-I crosses the blood-brain barrier, we could not assess the extent to which our measurements of circulating IGF-I receptor stimulating activity are related to actual IGF-I receptor stimulating activity in the brain (Reinhardt and Bondy, 1994). In addition, IGF-I has important autocrine and paracrine actions at the tissue level. However, IGF-I measured by the KIRA assay may not necessarily reflect IGF-I bioactivity at the local tissue level (Chen et al., 2003). Second, no total serum IGF-I levels were measured in our study, therefore we were unable to compare the relationship of IGF-I receptor stimulating activity and total IGF-I with dementia and to show that measuring IGF-I bioactivity with the IGF-I KIRA assay provides other insights about the role of IGF-I in dementia than the measurement of total IGF-I. Third, IGF-I receptor stimulating activity was assessed at the second follow-up visit of the Rotterdam Study, which might have led to survival effects in the study population which was included at baseline.

In conclusion, our current study sheds new light on the association between IGF-I signaling and the neuropathology of dementia, suggesting a threshold effect of IGF-I receptor stimulating activity moderated by ApoE genotype, since only for those with one or more copies of the ApoE-ε4 allele and in the lowest tertile of IGF-I receptor stimulating activity the risk of future dementia is decreased. Our study suggests that the ApoE-ε4 genotype modifies the relationship between IGF-I receptor stimulating activity and dementia and elevated IGF-I receptor stimulating activity levels mark a compensatory response to neuropathological changes associated with the ApoE-ε4 genotype. In line with the hypothesis that low IGF-I activity increases the risk of dementia, we found the ApoE-ε4 homozygotes, with a lifetime risk of AD of 80% (van der Lee et al., 2018), have the lowest IGF-I levels. This provides a genetic benchmark for the hypothesis that low IGF-I receptor stimulating activity is associated with an increased risk of AD. However, our findings require replication in other cohorts, reusing measures of IGF-I receptor stimulating activity rather than total IGF-I serum levels as putative predictors of dementia risk.

## Author Contributions

CD, MI, ES and SG contributed to the study concept and design. JJ and MB were responsible for the collection and assessment of IGF-I kinase receptor activation assay. SG and AS performed the statistical analyses. CD, JJ, MD and SG contributed to the interpretation of the results. SG drafted and revised the manuscript. All authors contributed to the critical revision of the manuscript. All authors approve the final manuscript as submitted and agree to be accountable for all aspects of the work.

## Conflict of Interest Statement

The authors declare that the research was conducted in the absence of any commercial or financial relationships that could be construed as a potential conflict of interest.

## Footnotes

^1^www.trialregister.nl

^2^www.who.int/ictrp/network/primary/en/
